# Tracking the Odysseys of Juvenile Schistosomes to Understand Host Interactions

**DOI:** 10.1371/journal.pntd.0000257

**Published:** 2008-07-16

**Authors:** Malcolm K. Jones, Sara Lustigman, Alex Loukas

**Affiliations:** 1 Division of Infectious Diseases, Queensland Institute of Medical Research, Herston, Queensland, Australia; 2 School of Veterinary Science, The University of Queensland, St Lucia, Queensland, Australia; 3 Laboratory of Molecular Parasitology, Lindsley F. Kimball Research Institute, New York Blood Center, New York, New York, United States of America; London School of Hygiene & Tropical Medicine, United Kingdom

## Background

The prospects confronting a “new-born” schistosome cercaria are formidable. That some of these microscopic helminths successfully negotiate the tortuous route from snail to human vasculature is a truly remarkable feature of adaptive biology. After escaping the birth pore of its parental sporocyst, a cercaria (at least we infer from studies of other digeneans [1]) swims and crawls through the snail body cavity before it burrows through a pre-formed escape tunnel to the aquatic environment. Once in that milieu, a cyclical suite of swimming behaviours positions the cercaria for its potential assault on the skin of an available host, should one appear. Upon skin penetration, the larva (now called a schistosomulum) sits within the skin for up to 72 hours before tracking to the lung, whereupon it re-enters a second static phase. This journey takes the organism through three distinct environments (five if we include the solid integuments of snail and human hosts), incorporates a wholesale remodelling of the surface membrane, and includes two poorly understood periods of relative immobility in the skin and the lung. Further development in the liver is required before the adults reach their ultimate destination in the vasculature of the intestine or bladder. How the juvenile stages of schistosomes negotiate these environments is of intense interest, not the least because protective immunity in schistosome infections, when it occurs, appears to be directed against the early intra-host stages, with the principal target being the lung stage schistosomulum [2].

There is much to be learnt about the first few weeks of cercarial establishment in the human host, but a detailed picture of the molecular and cellular events during this time has been difficult to obtain. The limitations arise partly because of the difficulties in accessing material from experimental or natural infection, and past technological limitations. Despite major advances in our understanding of parasite transformation (reviewed recently in [3]), and of the host–parasite interplay in early establishment [4], there remain many questions, the answers to which will undoubtedly guide discovery of novel targets for drugs and vaccines. Some of the questions that are still unanswered are:

- What is it that makes young schistosomes susceptible to some drugs (like artemether), while later developmental stages appear refractory [5]? In contrast, why is praziquantel ineffective against young schistosomula but effective against adult worms [6]?
- How do host genetics and parasite activities interplay to allow some people to respond effectively to parasite assault, while other people remain susceptible?
- What is the purpose of the periods of sequestration in the skin and the lung?
- What are the molecular and cellular events that are associated with the surface transformation?

The “-omics” explosion in biology has more recently also changed schistosomiasis research and enabled new ways of addressing some of the above issues in schistosome–host interactions. In recent issues of *PLoS Neglected Tropical Diseases*, two papers that use such advanced analytical tools have been published and both have advanced our understanding of the early development of schistosome parasites in their human hosts.

## Why Are Vaccinations with Radiation-Attenuated Cercariae Successful?

The first paper, by Dillon and colleagues [7], describes an in vitro model to explore the effects of radiation attenuation (RA) on gene expression in mechanically transformed *Schistosoma mansoni* cercariae. RA is a highly effective vaccine strategy, but the mechanisms of its success are not understood. Using a microarray designed to portray the transcriptome of lung-stage *S. mansoni* schistosomula [8], the authors were able to analyse global expression patterns of normal versus RA parasites at distinct time points for periods of up to 10 days, by which time the schistosomula would have migrated to the lungs. The authors demonstrated that although there are distinct differences between RA schistosomula and controls at various time points, the overriding effect of RA is that of non-specific disruption of gene expression, rather than enrichment of specific gene products. The effect of RA, then, is simply that of retarding migration and development of schistosomula, allowing time for the immune system to recognise key parasite molecules and to mount a protective response.

## Cercarial Interactions in the Skin

In a second paper, published in this issue, Hansell and colleagues [9] described proteomic analysis of *S. mansoni* cercarial secretions in human skin after cercarial penetration and transformation to schistosomula in vivo, as well as the host peptides that are present around the invading cercaria. These authors used a recently amputated limb as a model for invasion of human skin, which alone should ensure this article's prominence in the halls of “parasitological fame”. Although the authors readily admit that postmortem changes modify aspects of the skin structure, the model does serve as a picture of early molecular interactions of the transformed cercaria and its dermal environment. Newly penetrated larvae release a gamut of proteins from the acetabular glands, including cercarial elastases, serpins, paramyosin, and glutathione-*S*-transferases. Some of these molecules have been already recognised as the “old guards” of immunodominant vaccine candidates [10]. However, the present findings may help to further our understanding of the involvement of these molecules in host immune responsiveness. Other molecules released over the timeframe of analysis include tegument-associated antigens (Sm20.8) and other modulators of immune response, including a protein that belongs to the venom allergen-like protein family described for parasitic worms including the schistosomes [11]. Hansell et al. also performed proteomic analyses of the host dermal environment around the invading cercaria.

The human skin environment potentially includes skin factors associated with host responsiveness to the invasion of cercariae. The authors concurrently analysed secreted molecules of the skin to determine whether host proteins were either degraded by cercarial secretions or accumulated because of invasion. Comparison of skin exposed to cercariae, unexposed skin, and tissues punctured by needles to mimic the intra-dermal tunnels created by invading cercariae revealed that the physical barriers of epidermic, basement membrane, and dermis are augmented with host defence factors, such as complement components, immunoglobulins, and protease inhibitors. Data consistent with the complexing of the host protease inhibitors α-1-anti-trypsin and α-1-anti-chymotrypsin with cercarial elastase were inferred from the analyses. The presence of unmodified cercarial elastase perhaps indicates that this innate defence strategy is overwhelmed by invading cercariae. Complement components (C1 and C3) were less abundant in skin tissues exposed to cercariae. This may indicate that either the C3 pathway was activated in the skin in response to invading cercariae, or was degraded by cercarial proteases as a mechanism of immune evasion.

## Uniting the “omes” of Schistosomes (Schistosomics?)

Both contributions are inventive and demonstrate the power of combining rational experimental design with the more recently developed and available functional genomic tools to address questions related to crucial stages in establishment of schistosome infections. We now look forward to equally inventive strategies to explore other aspects of schistosome biology and development, given the complex transformation and “sojourn” of schistosomula in their mammalian hosts. The combination of these platforms, together with means for gene silencing or modification through RNAi and stable transfections [12], should provide substantial new information on parasite assault and host immunity. Microdissection methods will also enable analysis of the interplay within specific tissue microenvironments [13], particularly when coupled with transfected schistosomes expressing a fluorescent reporter [14]. As these new areas develop, fundamental knowledge of the secretome and surface structure of cercaria, the cell biology of surface modification of transforming schistosomula, and the repertoire of interactions among secreted parasite and tissue-specific molecules of the host, would be more easily achieved.
